# Effect of Potassium Deficiency on Physiological Responses and Anatomical Structure of Basil, *Ocimum basilicum* L.

**DOI:** 10.3390/biology11111557

**Published:** 2022-10-24

**Authors:** Houneida Attia, Fedia Rebah, Chayma Ouhibi, Muneera A. Saleh, Ashwaq T. Althobaiti, Khalid H. Alamer, Mouhiba Ben Nasri, Mokhtar Lachaâl

**Affiliations:** 1 Department of Biology, College of Sciences, Taif University, P.O. Box 11099, Taif 21944, Saudi Arabia; 2 Laboratory of Vegetable Productivity and Environmental Constraint LR18ES04, Department of Biology, University Tunis El Manar II, Tunis 1060, Tunisia; 3 Biological Sciences Department, Faculty of Science and Arts, King Abdulaziz University, P.O. Box 80200, Rabigh 21911, Saudi Arabia

**Keywords:** anatomical changes, growth, *Ocimum basilicum*, polyphenols, potassium deficiency

## Abstract

**Simple Summary:**

Potassium deficiency is a constraint that causes numerous dysfunctions at the plant level, notably a reduction in leaf growth, photosynthesis and production. Among the multiple physiological disturbances induced by potassium deficiency, some are manifested very early and others only when the deficiency is severe. Therefore, we studied the effect of insufficient potassium supply on physiological, biochemical and anatomical parameters in basil, *Ocimum basilicum*. Results revealed a decrease in root and leaf biomass production in K^+^-deficient plants in association with an inhibition of root elongation and a reduction in leaf expansion. Similarly, a decrease in photosynthetic pigments and variability in the response of phenolic compounds content was recorded, depending on the organ and the K^+^ concentration in the medium. Analysis of the anatomical structure of stems showed that exposure to potassium deficiency in basil decreased the diameter of these organs and the amount of lignin produced.

**Abstract:**

The aim of this study was to investigate the effect of a variable supply of potassium to culture medium on physiological and anatomical parameters (histological sections at the third internode) in basil, *Ocimum basilicum*. Thirty-four-day-old plants grown on basic nutrient medium were divided into four batches and grown on media with varying doses of potassium: 0.375 mM, 0.250 mM, 0.125 mM and 0 mM K^+^. After 64 days of culture, a final harvest was performed. The results showed that root and shoot growth in basil was decreased with decreased K^+^ concentration. This restriction was associated with a reduction in root elongation and leaf expansion, which was coupled with a decrease in chlorophyll and carotenoid contents. The estimation of electrolyte leakage reveals that this parameter was increased by potassium deficiency. With respect to total polyphenol and flavonoid contents, only the third leaf-stage extracts exhibited a decrease under low-K^+^ conditions. However, variability in response of phenolic compounds was recorded depending on the organ and the K^+^ concentration in the medium. Stem cross sections of potassium-deficient basil plants revealed a decrease in the diameter of these organs, which can be attributed to a restriction of the extent of different tissue territories (cortex and medulla), as well as by a reduction in cell size. These effects were associated with a decrease in the number of conducting vessels and an increase in the number of woody fibers.

## 1. Introduction

In their natural biotopes, plants are exposed to various stresses that significantly contribute to the restriction of their growth and development [1]. Potassium is an essential element that provides vital functions in plants and is required in large quantities [2]. Plants are able to take up this cation from external concentrations ranging from a few µM to tens of mM [3]. Cakmak [4] suggested that under stressful conditions, plants have a higher internal requirement for potassium. Therefore, selecting natural species that are resistant to potassium deficiency is an important strategy to overcome this constraint and consequently alleviate the effects of other environmental stresses, such as drought and salinity. Like most crop plants, basil has been shown to be sensitive to various abiotic stresses, such as drought [5], temperature [6], salinity [7,8] and heavy metals [9]. Nutritional deficiency affects vegetative development and photosynthetic capacity, which, in turn, can affect plant production [10]. Data on basil nutrition, including its nutritional requirements, as well as a description of the symptomatology of macronutrient deficiency, are scarce in the literature.

In plant physiology, potassium is the most important cation, not only in terms of quantity but also in terms of its many biochemical and physiological functions. However, it is the only major, indispensable element that is not a constituent of biomolecules. Its presence is necessary for cell turgidity and for maintenance of pH for many synthesis processes in the cytoplasm [11]. However, the most important feature of potassium is its rapid absorption by plant tissues. The amount of potassium taken up by a crop depends on crop species, the amount of potassium available in the soil and environmental conditions during the growing season [2].

Depending on the cell compartment, concentrations vary from 20 to 200 mM in the vacuole, which represents 90% of the cell volume, whereas in the cytoplasm potassium concentrations range between 100 and 200 mM [12]. The same concentration ranges are measured inside chloroplasts as in the cytoplasm. Once in the plant, potassium is involved in many processes. It is characterized by high mobility in the plant at all levels—in cells, in tissues and in vessels of raw or elaborated sap [12].

Nieves-Cordones et al. [12] proposed a model to understand why, depending on the level of deficiency experienced by the plant, different functions may be affected. This model is based on the observation that potassium is mainly located in vacuoles and in the cytoplasm and that these compartments have varying potassium levels.

The effects of K^+^ deficiency on biochemical functions can be summarized as an alteration in the synthesis of compounds essential for growth, i.e., sugars and proteins. However, the thresholds of deficiency that trigger these alterations are not precisely known.

K^+^ efficiency is defined as the ability of a plant to produce biomass under suboptimal potassium nutrition conditions. Different species and sometimes even varieties of the same species respond differently to the same level of K^+^ deficiency. Nieves-Cordones et al. [13] attributed this intra- and interspecific variability to the ability of plants to substitute other cations for K^+^ and to mobilize potassium at the cellular level and within the various organs, as well as to the nature of the root system and the specific functioning of each species.

One of the most important characteristics of secondary metabolite accumulation is it dependence on plant development stage; the organ, tissue and cells involved; and the type of environmental stress applied. The effect of various abiotic stresses, including salinity and nutritional deficiency, on antioxidant properties has been reported in several studies [2,14,15], most of which suggest that these environmental stresses induce a protective or restorative response in plants, including the production of antioxidants [2]. These factors also play an important role in determining total polyphenol content [16]. Because phenolic compounds provide information on the physiological state of plants, they can serve as an early “sensor” indicating the presence of stress. The development of secondary metabolites, such as polyphenolic compounds, represents an important tool for plants to overcome oxidative stress [17]. Hafsi et al. [18] established that K^+^ deficiency in *Sulla carnosa* modulates the composition of secondary metabolites and their antioxidant characteristics.

A large number of aromatic and medicinal plants have interesting biological properties that can be applied in various fields, such as medicine, pharmacy, cosmetology and agriculture, representing sources of active compounds. Known since ancient times, basil has powerful therapeutic properties owing to its various phytochemical constituents, as well as its essential oil. It is used as a traditional remedy for various ailments, such as poor digestion, nausea, migraines and insomnia. Its use also extends to the fields of perfumery and food, owing to its aroma.

In the present study, by integrating physiological (growth, mineral nutrition, etc.), biochemical (chlorophylls, carotenoids, polyphenols, flavonoids, etc.) and histological (stem cross sections) data, we aimed to study the effect of an insufficient K^+^ supply on aromatic and medicinal plants, such as basil, *Ocimum basilicum* var. Genovese, a commercial variety of basil characterized by its large leaves.

## 2. Materials and Methods

### 2.1. Growth Conditions

Basil seeds were disinfected with dilute sodium hypochlorite for a few minutes, then rinsed quickly with distilled water. Thereafter, seeds were placed in a beaker containing distilled water for two hours until imbibition. Then, they were germinated in Petri dishes lined with a double layer of filter paper soaked in distilled water at a rate of 25 seeds per dish.

Petri dishes containing 7-day-old *Ocimum basilicum* seedlings were placed in an air-conditioned culture room under a lighted ceiling, with all culture conditions controlled: light (16 h day/8 h night), temperature (22 °C day/18 °C night), effective radiation (200 µmol · m^−2^ · s^−1^) and hygrometry (60% humidity day/80% humidity night).

After 24 h, seedlings were transferred individually into 330 mL black plastic pots containing a nutrient solution (floating system). Mineral nutrition was provided by an aerated one-fourth-strength nutrient solution described by Arnon and Hoagland [19] containing 0.5 mM MgSO_4_, 0.25 mM KH_2_PO_4_, 1.25 mM Ca(NO_3_)_2_, 1.25 mM KNO_3_, 1 µM MnSO_4_, 0.5 µM ZnSO_4_, 10 µM H_3_BO_3_, 0.05 µM (NH_4_)Mo_7_O_24_ and 1.5 µM FeEDTA.

### 2.2. Application of Potassium Treatments

At the age of 34 days (beginning of the third leaf stage), plants were divided into 4 batches:

The first batch was the basic nutrient solution with 1.5 mM K^+^.

- To apply 1 mM K^+^, the KNO_3_ concentration was reduced from 1.25 mM to 0.75 mM, and 0.5 mM NaNO_3_ was added;
- To apply 0.5 mM K^+^, the KNO_3_ concentration was reduced from 1.25 mM to 0.25 mM, and 1 mM NaNO_3_ was added; and
- To apply total potassium deficiency, KNO_3_ salt (1.25 mM) and KH_2_PO_4_ salt (0.25 mM) were replaced by NaNO_3_ (1.25 mM) and NaH_2_PO_4_ (0.25 mM), respectively.

The solution was diluted 4 times, so the final concentrations of K^+^ in the medium were 0.375, 0.25, 0.125 and 0 mM K^+^.

Based on preliminary experiments, 0.375 mM K^+^ was found to be a suboptimal concentration at which no obvious deficiency symptoms were recorded. In this work, we will consider it as control.

After 30 days of treatment, plants were harvested and cut into roots, stems and leaves. Leaves of the 3rd and 4th stages and the remaining leaves were collected separately.

### 2.3. Analytical Techniques

Morphogenesis was monitored by counting the number of leaves and measuring root elongation with a ruler. Leaf area was measured using Optimas software.

Potassium content was determined at harvest by flame emission photometry and expressed in mmol · g^−1^ DW. Potassium quantities (expressed in mmol · organ^−1^) were also measured in roots and shoots.

Chlorophyll (Chl a and b) and carotenoid contents were estimated according to Lichtenthaler [20].

Electrolyte leakage was measured as described by Dionisio-Sese and Tobita [21].

Total flavonoid content was determined using the colorimetric method described by Zhishen et al. [22], which was based on the formation of a complex between flavonoids and aluminum trichloride.

Phenolic compounds were quantitatively determined using the Foline–Ciocalteu method [23], which involves the oxidation of this reagent to blue tungsten-molybdenum oxide; the intensity of the blue color informs the concentration of polyphenols in the extracts.

### 2.4. Preparation of Anatomical Sections

For anatomical study, transverse sections separated at the stem level (3rd node) and leaves (3rd leaf stage) from plant material previously preserved in 70% alcohol.

Freehand sections were taken for stems, whereas for leaves, a freezing microtome was used. Cross sections were then stained using the double staining method. Sections were incubated for 10 to 20 min in chlorex to remove cell contents and reserves. Thereafter, they were washed thoroughly with running water and rinsed with distilled water. Sections were then incubated in an acetic water bath (fixative) for 5 min and stained with carmine green for 30 min.

Carmine green is a mixture of carmine alum and methyl green. Carmine turns cellulosic and pectocellulosic structures (such as parenchyma, phloem and collenchyma) pink. Methyl green stains the lignified walls green and the suberized walls yellowish-green. After staining, sections were rinsed with distilled water and placed between a slide and coverslip in a drop of glycerin water and observed with a Leitz Orthoplan photomicroscope equipped with a camera

### 2.5. Methods of Statistical Analysis

Two methods of statistical analysis were used. The first was a simple comparison of means with Student’s *t* test. The other method was a one-way ANOVA with post hoc Duncan’s multiple-range test at *p* < 0.05.

## 3. Results

### 3.1. Effect of Potassium Deficiency on Plant Growth and Development

#### 3.1.1. Plant Aspect 

**Figure 1:**
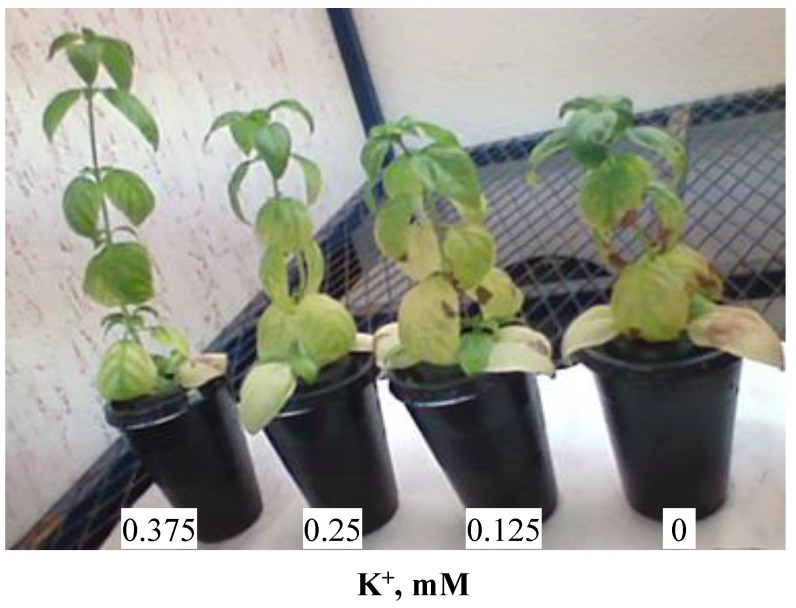
Morphology of 64-day-old *O. basilicum* plants after 30 days of culture in the presence of varying K^+^ concentrations.

Figure 1 shows the aspect of *O. basilicum* plants grown for 30 days in the presence of varying K^+^ concentrations: 0.375, 0.25, 0.125 and 0 mM. Growth decreased with the decreasing K^+^ concentration. Potassium deficiency was manifested by the appearance of straw-colored necrotic spots on leaf edges. A marginal discoloration also developed into necrosis. When deprived of K^+^, the oldest leaves were pale green in color and dried-out at the edges.

#### 3.1.2. Morphological Parameters 

The decrease in potassium concentrations (0.25, 0.125 and 0 mM K^+^) resulted in statistically significant decreases in leaf expansion of 37%, 45% and 54%, respectively, compared to the plants cultivated with 0.375 mM K^+^. The thickness of leaves in the third stage grown in the presence of 0.25, 0.125 and 0 mM K^+^ was increased by 45%, 78% and 46%, respectively, compared to the those grown with 0.375 mM K^+^ (Table 1).

Root elongation was significantly reduced at 0.125 mM K^+^ but significantly increased at 0 mM K^+^ as compared to the 0.375 mM K^+^ treatment (Table 1).

#### 3.1.3. Biomass Production

**Figure 2:**
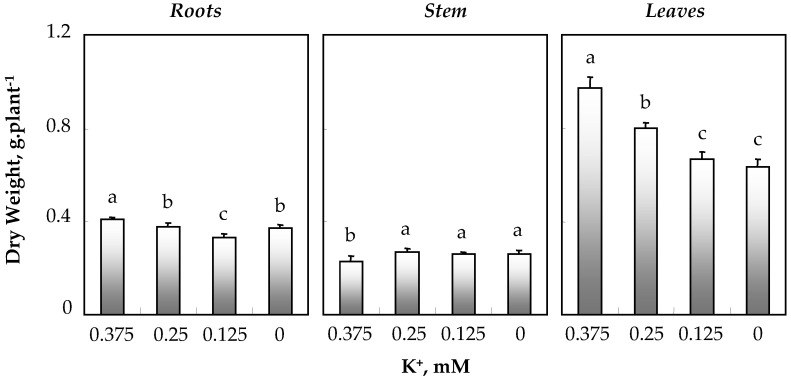
Dry weights of root, stem and all leaves of *O. basilicum* plants grown for 30 days in the presence of varying K^+^ concentrations (0.375; 0.25; 0.125 and 0 mM). Means of eight replicates and confidence intervals at the 5% level are presented. Bars with the same letters as indicated in the Figure are not significantly different according to Duncan’s test at *p* < 0.05.

Figure 2 shows that regardless of potassium concentration in the medium, the leaf total dry weight was much higher than that of roots and stems. A decrease in K^+^ concentration induced a reduction in leaf, total and root dry weights, with the effect on all leaves being more pronounced. In contrast, in stems, a slight significant increase in DW was observed at 0.25, 0.125 and 0 mM K^+^ (Figure 2).

**Figure 3:**
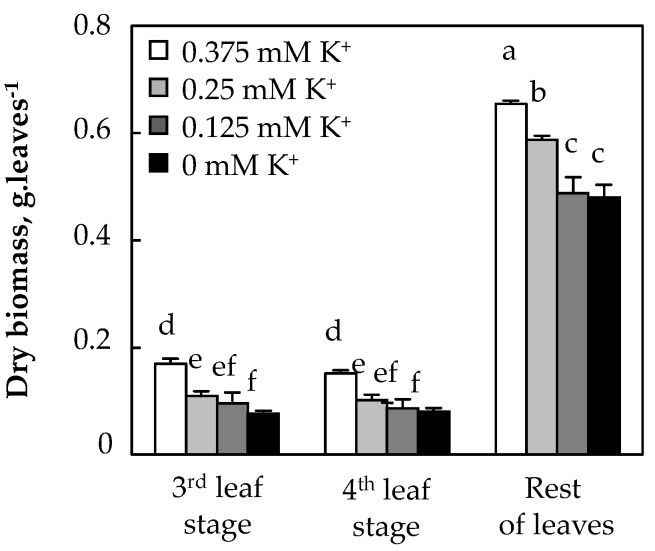
Dry weight of the third and fourth leaves and remaining leaves of *O. basilicum* plants. Cf. leg. Figure 2.

Because leaves determine the interception of radiation and are the main photosynthetic organs, the effects of potassium deficiency on growth in the leaf stage were considered in this work. Figure 3 shows that dry biomass of the third leaf stage decreased significantly with decreased K^+^ concentration in the medium. The same trend was observed for the fourth leaf stage and the rest of leaves, for which the DW was reduced in the total absence of K^+^, regardless of the position of the leaves—whether expanding, mature or senescent leaves (Figure 3).

#### 3.1.4. Potassium Concentrations and Amounts

**Figure 4:**
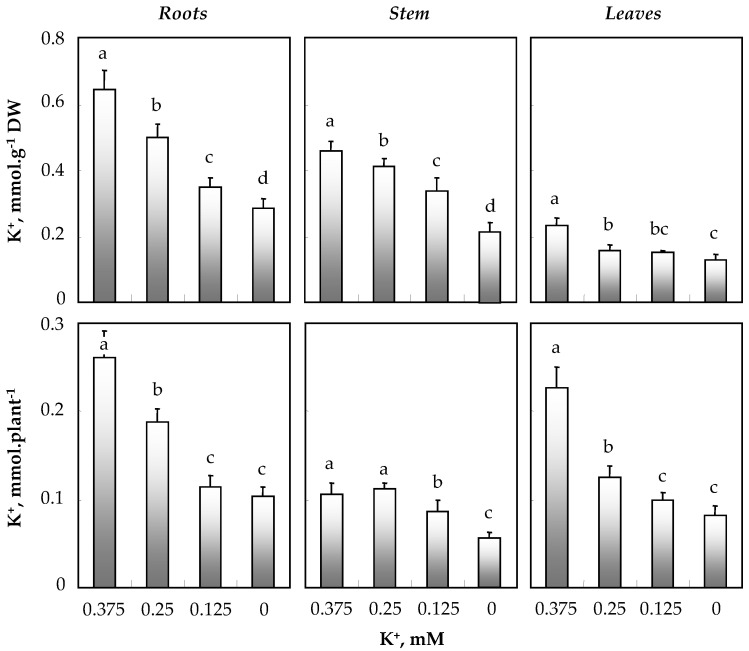
Variations in K^+^ concentrations and amounts in roots, stems and total leaves of 64-day-old *O. basilicum* plants grown for 30 days in the presence of varying K^+^ concentrations (0.375, 0.25, 0.125 and 0 mM). Cf. leg. Figure 2.

Figure 4 shows that in the medium with the highest K^+^ level (0.375 mM K^+^), K^+^ concentrations were much higher in the roots (0.6 mmol · g^−1^ DW) and, to a lesser extent, in the stems (0.5 mmol · g^−1^ DW) than in the leaves (0.2 mmol · g^−1^ DW). A decrease in potassium concentration in the medium led to a more pronounced disturbance of potassium supply in leaves and roots than in stems.

Like K^+^ concentrations, the amounts of K^+^ (mmol · plant^−1^) showed similar variations for roots and leaves that accumulated large K^+^ amounts. These amounts decreased with decreased K^+^ concentration in the medium. As for stem K^+^ amounts, they were reduced at 0.125 and 0 mM K^+^, with a decrease of ~50% at 0 mM K^+^ compared to plants supplied with 0.375 mM K^+^ (Figure 4). These results show that the reduction in leaf growth in the presence of low concentrations of potassium in the medium seems to be the result of the decrease in the amounts of potassium in these photosynthetic organs.

Given that different stages of leaf development show similar evolutionary trends with respect to all studied parameters of growth and potassium nutritional status and according to the treatments applied in the medium, the additional analyses and other parameters studied in this work focused particularly on the third leaf stage.

### 3.2. Effect of K^+^ Concentration in the Medium on Chlorophylls and Carotenoids

The progressive decrease in K^+^ in the medium led to a decrease in Chl *a* and *b* contents (Table 2). The same trend was observed for total chlorophylls, although the Chl *a*/*b* ratio remained stable at 0.375, 0.25 and 0.125 mM K^+^, with a decrease at 0 mM K^+^. Carotenoid levels showed a similar pattern to those of chlorophylls. The decrease in these levels reached 45% at 0 mM K^+^. The Chl/Car ratio remained unchanged under conditions of potassium deficiency.

### 3.3. Effect of K^+^ Deficiency on Membrane Integrity

**Figure 5:**
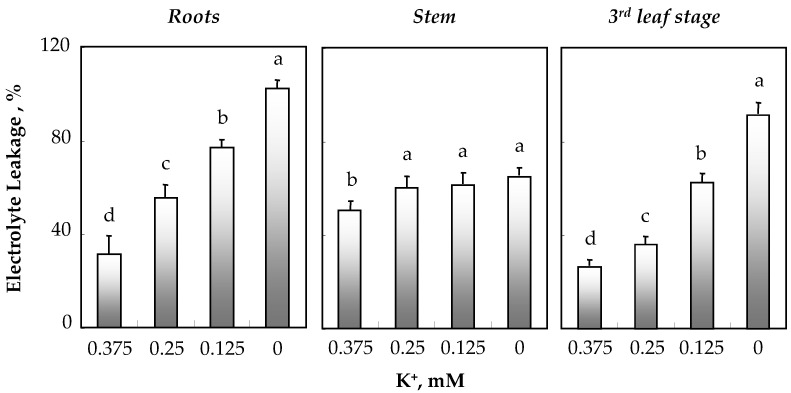
Variations in electrolyte leakage (EL) in roots, stem and the third leaf stage of 64-day-old *O. basilicum* plants grown for 30 days in the presence of varying K^+^ concentrations (0.375, 0.25, 0.125 and 0 mM). Cf. leg. Figure 2.

Membrane integrity was estimated in roots, stems and leaves (third leaf stage) of *O. basilicum* by measuring electrolyte leakage (EL) after 34 days of treatment with varying concentrations of K^+^ in the medium. Figure 5 shows an increase in EL in both roots and leaves as K^+^ concentration decreased in the medium. In leaves (precisely in the third leaf stage), EL increased by 1.5-, 2.5- and 3.5-fold at 0.25, 0.125 and 0 mM K^+^, respectively. The stems of plants supplied with 0.25, 0.125 and 0 mM K^+^ exhibited a similar slight increase in EL of about 16% compared to that of plants grown in the presence of 0.375 mM K^+^ (Figure 5).

### 3.4. Effect of K^+^ Concentration in the Medium on Total Polyphenols and Flavonoids

**Figure 6:**
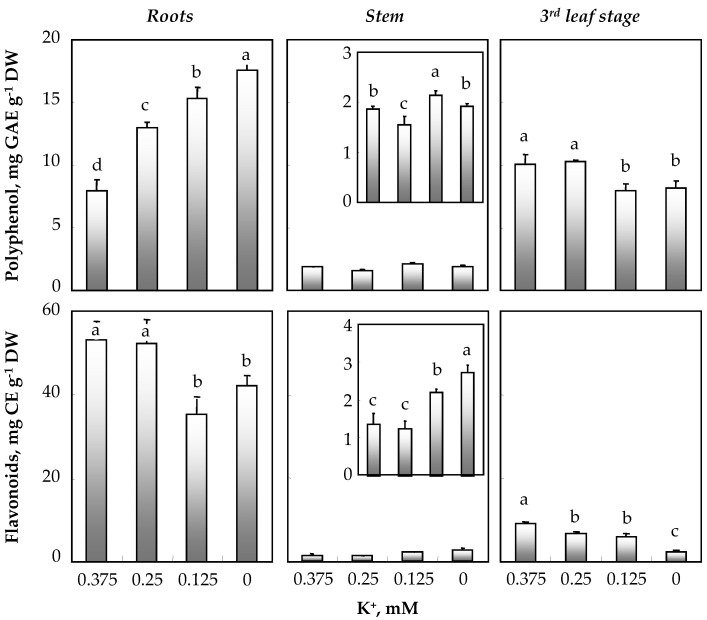
Variations in polyphenol and flavonoid contents in roots, stems and the third leaf stage of 64-day-old *O. basilicum* plants grown for 30 days in the presence of varying K^+^ concentrations (0.375; 0.25; 0.125 and 0 mM). Means of six replicates and confidence intervals at the 5% level are presented. Bars with the same letters as indicated in the Figure are not significantly different according to Duncan’s test at *p* < 0.05.

Figure 6 shows that regardless of potassium concentration in the medium, roots and leaves were the richest organs in polyphenols compared to stems. In roots, an increasing gradient of polyphenol production was observed, reaching 18.1 mg GAE g^−1^ DW under conditions of total K^+^ deprivation. In stems and in contrast to roots, polyphenol contents decreased in the presence of 0.25 mM K^+^ and increased in the presence of 0.125 mM K^+^ as compared to the 0.375 mM K^+^ treatment. As for leaves and, more precisely the third leaf stage, the production of polyphenols showed a statistically significant decrease exclusively at the lowest concentrations of K^+^ in the medium (0.125 and 0 mM) (Figure 6).

Figure 6 clearly shows that the root flavonoid content in plants supplied with 0.375 mM K^+^ was superior to that in their stems and leaves. Root flavonoid contents were 38 times higher than those of stems. The extracts from the roots and leaves of plants grown in the presence of low K^+^ concentrations showed lower levels of total flavonoids than plants cultivated in the presence of 0.375 mM K^+^. For stems and in contrast to other organs, the contents of these phenolic compounds increased at 0.125 mM K^+^ and were doubled in total absence of K^+^.

### 3.5. Effect of K^+^ Deficiency on the Anatomical Structure of O. Basilicum Stems

One of the objectives of our anatomical study was to investigate the effects of potassium deficiency on the structure of stems. To this end, transverse sections of this organ were separated at the level of the third node.

#### 3.5.1. Structure of the Stem (0.375 mM K^+^)

**Figure 7:**
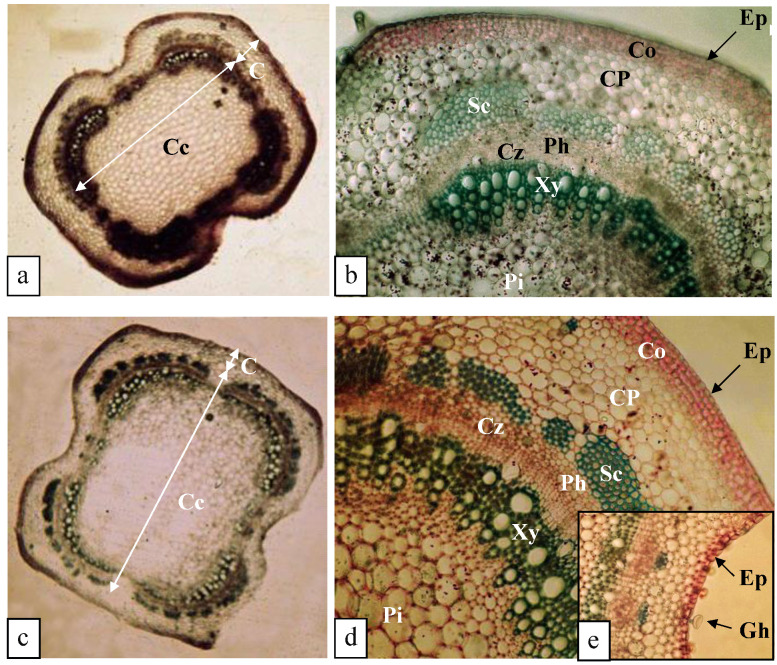
Transverse sections of *O. basilicum* stems of 64-day-old plants grown for 30 days in the presence of varying K^+^ concentrations. Fixation in F.A.A. and carmine-green staining. (**a**) Overall view of the stem at 0.375 mM K^+^ (×50). (**b**) Detail of Figure 7a (×231). (**c**) Overall view of the stem at 0.25 mM K^+^ (×50). (**d**) Detail of Figure 7c (×235). (**e**) Detail of Figure 7d (×398). Abbreviations: Cz—cambial zone; Cc—central cylinder; C—cortex; Co—collenchyma; CP—cortical parenchyma; Ep—epidermis; Fi—fibers; Gh—glandular hairs; Sc—sclerotized tissue; Ph—phloem; Pi—pith; Xy—xylem.

A cross section of the stem shows a reduced cortex (C) and a developed central cylinder (Cc) (Figure 7a). The cortex was bounded externally by a unistratified epidermis (Ep) with a cutinized outer wall. The epidermal cells were more or less isodiametric, with some of them extended by glandular hairs (Gh). The cortex was supported by an angular collenchyma (Co) formed by four to five thick-walled cell layers lined by a cortical parenchyma (CP) with meatus made up of five to six layers of more or less rounded, thin-walled cells. At the level of the central cylinder (Cc), the vascular bundles formed a continuous pachyte at the level of the cambial zone (Cz) surrounding a central pith (Pi) with large cells. This cylinder was surrounded externally by sclerotized tissue (Sc) (Figure 7b).

#### 3.5.2. Structure of the Stem (0.25 mM K^+^)

The anatomical structure of the stem of potassium-deficient plants was similar to that of the stems of plants grown at 0.375 mM K^+^, with the collenchyma (Co) containing four to five layers of cells but with thicker walls. However, an increase in stem diameter (252 µm) was observed due to an increase in the number of cortical cells (the cortical parenchyma (CP) was composed of 8–10 cell layers) (Figure 7c–e).

#### 3.5.3. Structure of the Stem (0.125 mM K^+^)

**Figure 8:**
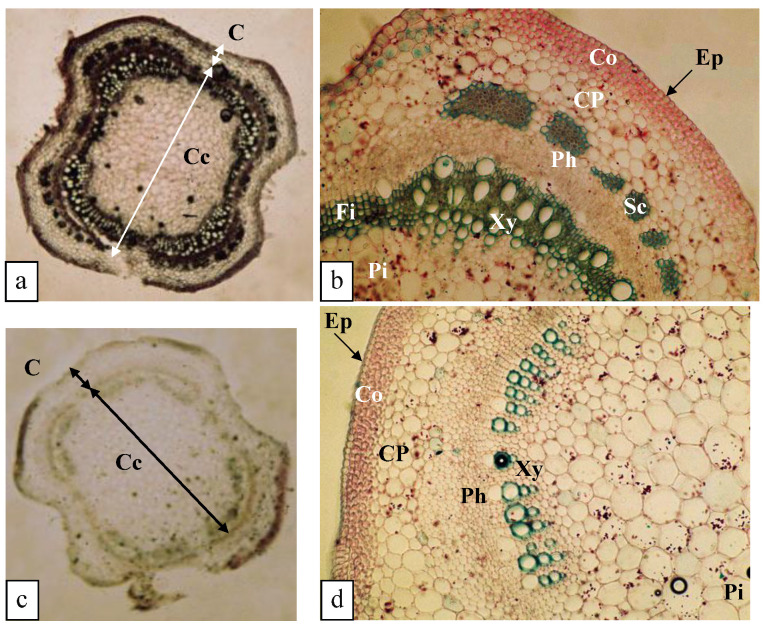
Transverse sections of *O. basilicum* stems of 64-day-old plants grown for 30 days in the presence of varying K^+^ concentrations. Fixation in F.A.A. and carmine green staining. (**a**) Overall view of the stem at 0.125 mM K^+^ (×50). (**b**) Detail of Figure 8a (×223). (**c**) Overall view of the stem at 0 mM K^+^ (×50). (**d**) Detail of Figure 8c (×211). Cf. leg. Figure 7.

Compared to the stems of plants supplied with 0.375 mM K^+^, a number of changes in the anatomical structure were noted, as follows (Figure 8a,b): (*i*) a decrease in the extent of the sclerotized tissue (Sc) surrounding the central cylinder (Cc); and (*ii*) at the level of the xylem (Xy), a decrease in the number of vessels associated with an increase in the number of fibers (Fi) (Figure 8a,b).

#### 3.5.4. Structure of the Stem (0 mM K^+^)

Stem transverse sections showed a decrease in stem diameter from 247 µm in plants supplied with 0.375 mM K^+^ to 239 µm in plants grown in the absence of K^+^ (Figure 8c). With respect to the anatomical structure, it underwent some modifications: at the level of the cortex (C), sclerenchymatous tissue (Sc) disappeared, and at the level of the central cylinder (Cc), a decrease in the number of conducting vessels was observed. There was also an absence of lignification of the woody parenchymatous tissue, which became cellulosic, as well as an increase in the diameter of the pith (Pi) (Figure 8d).

## 4. Discussion

The results presented above illustrate the effects of potassium deficiency on the investigated plants, as demonstrated by changes in physiological parameters and histological sections of *Ocimum basilicum* var. Genovese.

After 30 days of treatment, the cultivation of basil on a potassium-depleted medium led to a reduction in plant growth and to the appearance of chlorosis, which increased with decreasing K^+^ concentration in the medium (Figure 1). Our results are in agreement with those reported by dos Santos Sarah et al. [24], who showed that potassium deficiency resulted in chlorosis in the oldest leaves of bean plants cultivated in a hydroponic system. Other researchers found that K^+^ deficiency was manifested by the presence of brown spots on old leaves [25]. The symptoms of potassium deficiency are not characteristic. Growth reduction is the first consequence of insufficient potassium supply. As the deficiency becomes more severe, morphological symptoms appear. Leaf margins turn yellow and dry out and necroses appear [25].

Our results show also that potassium deficiency affected the growth of vegetative organs. In contrast to the stems, roots and leaves were more sensitive to potassium deficiency. The sensitivity of roots to potassium deficiency may be due to a reduction in lateral root growth as a result of increased expression of genes related to ethylene production, as well as its production, as suggested by Hetherington et al. [26]. In addition to the reduction in lateral root formation, a decrease in root length can also influence biomass production in these organs, especially under conditions of very limited K^+^ concentrations in the medium (0.125 mM). We showed that in addition to its depressive effect on biomass production, potassium deficiency affected the distribution of biomass between shoots (S) and roots (R) (data not shown). A significant decrease in the S:R ratio was observed upon potassium deprivation, which can be explained by a greater reduction in the growth of aboveground parts (mainly leaves) compared to belowground parts. In barley, Coffey et al. [27] showed a decrease in the S:R ratio as K^+^ availability was limited in the medium, suggested that this was due to the role of this cation in the transport of photoassimilates from shoots to roots via the phloem. Therefore, potassium deficiency resulted in a limited transport of photoassimilates from photosynthetic organs to roots, leading to an accumulation of carbohydrates in shoots and thus an increase in the S:R ratio. However, this proposal does not explain the decrease in the S:R ratio caused by potassium deficiency t has observed in other works [28]. In basil, no significant variations in the S:R ratio were recorded at 0.25 and 0.125 mM K^+^, suggesting that both organs were affected in the same way.

Potassium is a macronutrient involved in several processes in plants, such as photosynthesis, enzyme activation and osmoregulation, as well as the formation of carbohydrates, nucleic acids and proteins [29]. Under K^+^-limiting conditions, plant survival depends not only on the efficiency of K^+^ uptake but also on the efficiency of K^+^ utilization [30]. A reduction in K^+^ acquisition, as well as a lack of K^+^ in the medium is compensated by conservation for biomass production. Potassium is highly mobile in phloem sap, in which it can represent up to 80% of all cations, suggesting that redistribution of K^+^ from older leaves to young, growing organs could contribute to the improvement of its utilization efficiency [31].

Chlorophylls and carotenoids are essential for normal photosynthetic activity in green leaves. In this study, we measured the levels of these two types of pigments in *O. basilicum* in the presence of low K^+^ concentrations in the medium in order to investigate possible characteristic changes under such limiting conditions. Our results showed that the content of chlorophyll and carotenoid pigments dropped progressively with low K^+^ concentrations in the medium, except under conditions of potassium deprivation. These results are comparable to those reported by dos Santos Sarah et al. [24], who showed that potassium deficiency in the growing medium decreased chlorophyll content in bean plants. The apparent coordination of the decrease in carotenoid content with that of chlorophylls suggests that chlorophyll and carotenoid biosynthetic pathways, which both occur in the plastids [32], are synchronously regulated in *O. basilicum* leaves. This was also observed in several other species, such as sorghum [33].

We studied the effect of potassium deficiency on the production of some secondary metabolites, such as polyphenols and flavonoids, in *Ocimum basilicum* organs. Our results show a strong variability in the content of these biologically active compounds depending on the K^+^ concentrations in the medium the considered organ. In the present study, K^+^ deficiency decreased leaf polyphenol and flavonoid contents (Figure 6). Similar results were observed by Nguyen et al. [34]; however, in contrast with what was observed in *Sulla carnosa* [16] and in *Hordeum vulgare* [35], they [34] showed that low concentrations of K^+^ in the medium decreased the concentration of phenolic compounds in basil leaves. In contrast, high doses of K^+^ (5 mM) led to an increase in these secondary metabolites, as well as their antioxidant capacity.

According to Kanai et al. [36], four days of potassium deprivation culture were induced a decrease in stem diameter in tomato. In the present study, we observed a slight increase in stem diameter in plants cultivated at 0.25 mM K^+^ due to the increase in parenchymal cells. However, at 0.125 mM K^+^ and 0 mM K^+^, a decrease in stem diameter was revealed as a consequence of a decrease in cell number, in particular in plants grown in the total absence of K^+^. In addition to these adaptive anatomical changes under stressful conditions, plants modify the amount of produced lignin, and some studies clearly showed that lignin content increased in response to abiotic stresses [37]. Because lignification is essential for the structural integrity of plant cell walls and crucial for plant development, plants respond to stresses by increasing lignin production in specific tissues involved in conduction (xylem) or support tissues (sclerenchyma) [38].

## 5. Conclusions

The results obtained in the present study revealed a decrease in root and leaf biomass production in K^+^-deficient plants in association with an inhibition of root elongation and a reduction in leaf expansion. Similarly, a decrease in photosynthetic pigments in leaves of potassium-deficient plants was recorded, suggesting that changes in carotenoid contents were mainly controlled by endogenous factors related to chlorophyll synthesis. Leaf polyphenol and flavonoid contents were decreased at low K^+^ concentrations in the medium, strongly supporting the idea that these two phenolic compounds play an important physiological role in the tolerance of *Ocimum basilicum* to potassium deficiency and, in particular, against the oxidative damages it induces. Stem anatomical structure showed that exposure to potassium deficiency in basil decreased the diameter of these organs, as well as the amount of lignin produced, compared to plants grown in the presence of 0.375 mM K^+^.

## Figures and Tables

**Table 1:** Effects of potassium deficiency on root elongation, leaf area and thickness in 64-day-old *O. basilicum* plants. Measurements carried out after 30 days of treatment in the presence of varying K^+^ concentrations (0.375; 0.25; 0.125 and 0 mM). Means of eight replicates and confidence intervals at the 5% level are presented. Values with the same letters as indicated in the Table are not significantly different according to Duncan’s test at *p* < 0.05.

	K^+^, mM			
**Parameter**	**0.375**	**0.25**	**0.125**	**0**
Root elongation, cm	29 ± 2 ^b^	28 ± 1 ^b^	26 ± 1 ^c^	32 ± 1 ^a^
Leaf area, cm^2^ · plant^−1^	25 ± 2 ^a^	16 ± 1 ^b^	14 ± 1 ^c^	11 ± 1 ^d^
Thickness, cm	0.02 ^c^	0.03 ^b^	0.04 ^a^	0.03 ^b^

**Table 2:** Chlorophyll a, b, total and carotenoid contents (mg · g^−1^ FW) of third-leaf-stage leaves of 64-day-old *O. basilicum* plants. Measurements were carried out after 30 days of treatment in the presence of varying K^+^ concentrations (0.375, 0.25, 0.125 and 0 mM). Means of eight replicates and confidence intervals at the 5% level are presented. Values with the same letters as indicated in the Table are not significantly different according to Duncan’s test at *p* < 0.05.

	K^+^, mM			
**Pigment**	**0.375**	**0.25**	**0.125**	**0**
Chlorophyll a	19.8 ± 1.0 ^a^	14.1 ± 1.2 ^b^	10.6 ± 1.2 ^c^	7.2 ± 1.4 ^d^
Chlorophyll b	7.7 ± 0.7 ^a^	6.4 ± 0.5 ^b^	4.7 ± 0.5 ^c^	4.2 ± 0.6 ^c^
Chlorophyll total	27.0 ± 1.2 ^a^	18.3 ± 1.7 ^b^	14.3 ± 1.7 ^c^	13.3 ± 1.8 ^c^
Chl a/b	2.6 ± 0.2 ^a^	2.2 ± 0.1 ^a^	2.3 ± 0.5 ^a^	1.7 ± 0.2 ^b^
Carotenoid (Car)	6.1 ± 0.2 ^a^	4.4 ± 0.3 ^b^	4.2 ± 0.5 ^b^	3.4 ± 0.3 ^c^
Chl/Car	4.5 ± 0.3 ^a^	4.2 ± 0.6 ^a^	3.5 ± 0.8 ^a^	4.0 ± 0.5 ^a^

## Data Availability

Not applicable.

## References

[1] Hafsi C., Romero-Puertas M.C., del Rio L.A., Sandalio L.M., Abdelly C. (2010). Differential antioxidative response in barley leaves subjected to the interactive effects of salinity and potassium deprivation. Plant Soil.

[2] Hasanuzzaman M., Borhannuddin-Bhuyan M.H.M., Nahar K., Shahadat-Hossain M., Al Mahmud J., Shahadat-Hossen M., Chowdhury-Masud A.A., Moumita Fujita M. (2018). Potassium: A Vital Regulator of Plant Responses and Tolerance to Abiotic Stresses. Agronomy.

[3] Marschner P. (2012). Marschner’s Mineral Nutrition of Higher Plants.

[4] Cakmak I. (2005). The role of potassium in alleviating detrimental effects of abiotic stresses in plants. J. Plant Nutr. Soil Sci..

[5] Damalas C.A. (2019). Improving drought tolerance in sweet basil (*Ocimum basilicum*) with salicylic acid. Sci. Hortic..

[6] Barickman T.C., Olorunwa O.J., Sehgal A., Walne C.H., Reddy K.R., Gao W. (2021). Yield, Physiological Performance, and Phytochemistry of Basil (*Ocimum basilicum* L.) under Temperature Stress and Elevated CO_2_ Concentrations. Plants.

[7] Attia H., Ouhibi C., Ellili A., Msilini N., Bouzaïen G., Karray N., Lachaâl M. (2011). Analysis of salinity effects on basil leaf surface area, photosynthetic activity, and growth. Acta Physiol. Plant..

[8] Attia H., Alamer K., Algethami B., Ellouzi H., Lachaâl M. (2020). Effects of growth and salinity on some morphological parameters, pigment and flavonoid concentrations in basil. Agrochimica.

[9] Zahedifar M., Moosavi A.A., Shafigh M., Zarei Z., Karimian F. (2016). Cadmium accumulation and partitioning in *Ocimum basilicum* as influenced by the application of various potassium fertilizers. Arch. Agron. Soil Sci..

[10] Borges B.M.M.N., Flores R.A., Almeida H.J., Moda L.R., Prado R.M. (2016). Macronutrient Omission and the Development and Nutritional Status of Basil in Nutritive Solution. J. Plant Nutr..

[11] Wang M., Zheng Q., Shen Q., Guo S. (2013). The critical role of potassium in plant stress response. Int. J. Mol. Sci..

[12] Nieves-Cordones M., Ródenas R., Lara A., Martínez V., Rubio F. (2019). The combination of K^+^ deficiency with other environmental stresses: What is the outcome?. Physiol. Plant..

[13] Nieves-Cordones M., Martínez V., Benito B., Rubio F. (2016). Comparison between Arabidopsis and rice for main pathways of K^+^ and Na^+^ uptake by roots. Front. Plant Sci..

[14] Qi D., Zhao X., Le X., Jiang C., Wang X., Yi H., Wang J., Yu H. (2019). Effects of potassium deficiency on photosynthesis, chloroplast ultrastructure, ROS, and antioxidant activities in maize (*Zea mays* L.). J. Integr. Agric..

[15] de Oliveira R.L.L., de Mello Prado R., Felisberto G., Checchio M.V., Gratão P.L. (2019). Silicon mitigates manganese deficiency stress by regulating the physiology and activity of antioxidant enzymes in sorghum plants. J. Soil Sci. Plant Nutr..

[16] Hafsi C., Bettaib J., Falleh H., Zorrig W., Ksouri R., Abdelly C., Debez A. (2021). Phenolic accumulation and related antioxidant capacity in stems and roots of the Tunisian extremophile *Sulla arnosa* as influenced by potassium application under salinity stress. Arab. J. Geosci..

[17] Šamec D., Karalija E., Šola I., Bok V.V., Salopek-Sondi B. (2021). The role of polyphenols in abiotic stress response: The influence of molecular structure. Plants.

[18] Hafsi C., Falleh H., Saada M., Rabhi M., Mkadmini K., Ksouri R., Abdelly C., Smaoui A. (2016). Effects of potassium supply on growth, gas exchange, phenolic composition, and related antioxidant properties in the forage legume *Sulla carnosa*. Flora.

[19] Arnon D.I., Hoagland D.R. (1940). Crops production in artificial solution and in soils with special reference to factors affecting yields and absorption of inorganic nutrients. Soil Sci..

[20] Lichtenthaler H.K. (1987). Chlorophylls and carotenoids: Pigments of photosynthetic biomembranes. Methods Enzy..

[21] Dionisio-Sese M.L., Tobita S. (1998). Antioxidant responses of rice seedlings to salinity stress. Plant Sci..

[22] Zhishen J., Mengcheng T., Jianming W. (1999). The determination of flavonoids contents in mulberry and their scavenging effects on superoxide radicals. J. Food Chem..

[23] Cicco N., Lanorte M., Paraggio M., Viggiano M. (2009). A reproducible, rapid and inexpensive Folin-Ciocalteu micro-method in determining phenolics of plant methanol extracts. Microchem. J..

[24] dos Santos Sarah M.M., de Mello Prado R., de Souza Júnior J.P., Teixeira G.C.M., dos Santos Duarte J.C., de Medeiros R.L.S. (2021). Silicon Supplied via Root or Leaf Relieves Potassium Deficiency Effects in Common Bean. Sci Rep..

[25] Marathe R.A., Murkute A.A., Dhinesh B.K. (2016). Mineral Nutrient Deficiencies and Nutrient Interactions in Pomegranate. Natl. Acad. Sci. Lett..

[26] Hetherington F.M., Kakkar M., Topping J.F., Lindsey K. (2021). Gibberellin signaling mediates lateral root inhibition in response to K^+^-deprivation. Plant Physiol..

[27] Coffey O., Bonfield R., Corre F., Althea Sirigiri J., Meng D., Fricke W. (2018). Root and cell hydraulic conductivity, apoplastic barriers and aquaporin gene expression in barley (*Hordeum vulgare* L.) grown with low supply of potassium. Ann. Bot..

[28] Peuke A.D., Jeschke W.D., Hartung W. (2002). Flows of elements, ions and abscisic acid in *Ricinus communis* and site of nitrate reduction under potassium limitation. J. Exp. Bot..

[29] Demidchik V. (2014). Mechanisms and physiological roles of K^+^ efflux from root cells. J. Plant Physiol..

[30] Cui J., Abadie C., Carroll A., Lamade E., Tcherkez G. (2019). Responses to K deficiency and waterlogging interact via respiratory and nitrogen metabolism. Plant Cell Environ..

[31] Sustr M., Soukup A., Tylova E. (2019). Potassium in root growth and development. Plants.

[32] Quian-Ulloa R., Stange C. (2021). Carotenoid biosynthesis and plastid development in plants: The role of light. Int. J. Mol. Sci..

[33] Chen D., Cao B., Qi L., Yin L., Wang S., Deng X. (2016). Silicon-moderated K-defciency-induced leaf chlorosis by decreasing putrescine accumulation in sorghum. Ann. Bot..

[34] Nguyen P.M., Kwee E.M., Niemeyer E.D. (2010). Potassium rate alters the antioxidant capacity and phenolic concentration of basil leaves (*Ocimum basilicum* L.). Food Chem..

[35] Benslima W., Zorrig W., Bagues M., Abdelly C., Hafsi C. (2021). Silicon mitigates potassium deficiency in *Hordeum vulgare* by improving growth and photosynthetic activity but not through polyphenol accumulation and the related antioxidant potential. Plant Soil.

[36] Kanai S., Moghaieb R.E., El-Shemy H.A., Panigrahi R., Mohapatra P.K., Ito J., Nguyen N.T., Saneoka H., Fujita K. (2011). Potassium deficiency affects water status and photosynthetic rate of the vegetative sink in green house tomato prior to its effects on source activity. Plant Sci..

[37] Cabane M., Afif D., Hawkin S. (2012). Lignins and Abiotic Stresses. Advances in Botanical Research.

[38] Liu Y., Yin Q., Dai B., Wang K.L., Lu L., Qaseem M.F., Wang J., Li H., Wu A.M. (2021). The key physiology and molecular responses to potassium deficiency in *Neolamarckia cadamba*. Ind. Crops Prod..

